# Unraveling the web of defense: the crucial role of polysaccharides in immunity

**DOI:** 10.3389/fimmu.2024.1406213

**Published:** 2024-10-25

**Authors:** Yu Shen, Hongbo Zhao, Xuefeng Wang, Shihao Wu, Yuliang Wang, Chaoxing Wang, Yu Zhang, Hong Zhao

**Affiliations:** ^1^ College of Pharmacy, Jiamusi University, Jiamusi, China; ^2^ College of Rehabilitation Medicine, Jiamusi University, Jiamusi, China

**Keywords:** polysaccharides, immunity, immune regulation, natural polysaccharides, gut microbiota, immunostimulatory activity

## Abstract

The great potential of polysaccharides in immunological regulation has recently been highlighted in pharmacological and clinical studies. Polysaccharides can trigger immunostimulatory responses through molecular identification, intra- and intercellular communication via direct or indirect interactions with the immune system. Various immunostimulatory polysaccharides or their derivative compounds interacts at cellular level to boost the immune system, including arabinogalactans, fucoidans, mannans, xylans, galactans, hyaluronans, fructans, pectin and arabinogalactans, etc. These natural polysaccharides are derived from various plants, animals and microbes. A unique structural diversity has been identified in polysaccharides, while monosaccharides and glucosidic bonds mainly confer diverse biological activities. These natural polysaccharides improve antioxidant capacity, reduce the production of pro-inflammatory mediators, strengthen the intestinal barrier, influence the composition of intestinal microbial populations and promote the synthesis of short-chain fatty acids. These natural polysaccharides are also known to reduce excessive inflammatory responses. It is crucial to develop polysaccharide-based immunomodulators that could be used to prevent or treat certain diseases. This review highlights the structural features, immunomodulatory properties, underlying immunomodulatory mechanisms of naturally occurring polysaccharides, and activities related to immune effects by elucidating a complex relationship between polysaccharides and immunity. In addition, the future of these molecules as potential immunomodulatory components that could transform pharmaceutical applications at clinical level will also be highlighted.

## 1 Introduction

As the largest naturally occurring polymer in nature, polysaccharides exhibited a wide variety of structural and functional forms (1, 2). Advances and easy access to certain analytical tools help in the identification and quantification of polysaccharides, thereby supporting in-depth studies to determine the biological activities of polysaccharides. The polysaccharides shared certain structural features, just like other polymers such as proteins and nucleic acids (DNA, RNA), in which monosaccharide residues were linked together by glycosidic bonds (3). There are major differences in the structure of polysaccharides, which perform numerous biological functions at the cellular and tissue levels. In oligosaccharides and polysaccharides, the monosaccharide molecules can reassemble multiple times to form countless linear or branched structures, each with different functions. Polysaccharides, like proteins, exhibit the highest structural diversity ever known, with cosmopolitan distribution in nature, where they perform a variety of functions, as building blocks in cell membranes, energy storage, cell recognition, differentiation, proliferation and control of cell signaling (4–7). The biological properties of polysaccharides have attracted increasing attention in biochemistry and medicine in recent decades.

Polysaccharides exhibit a wide range of biological activities, including antioxidant, anti-inflammatory, antidiabetics, gut microbiota regulation and immunomodulatory effects (8, 9). Polysaccharides have emerged as significant players in the field of immunology due to enhancing the innate and adaptive immune response of the host. Polysaccharides stimulate immune cells via specific receptors, for example in necrophage and macrophage cells via bonds between functional groups of polysaccharides and groups of molecules on the cell surface. As soon as the polysaccharides bind to membrane receptors in the defense cells, the signaling pathways are activated and a cycle of biochemical processes begins, which leads to a positive regulation of gene expression in the ribosomes and initiates protein production. Moreover, polysaccharides influence immunity by modulating cytokines that have pro- and anti-inflammatory effects. They activate macrophages via signaling pathways such as TLRs and NF-κB and thus improve immunological functions. The ability of polysaccharides to enhance macrophage function is a crucial aspect of their immunomodulatory potential, as these cells are pivotal in the body’s defense mechanisms. Certain polysaccharides, such as those from Tinospora cordifolia, have been shown to increase macrophage activity, while Bupleurum polysaccharides have been shown to reduce inflammation (10). T lymphocytes produce cytokines that influence inflammatory responses, such as IL-10 and IL-4 (Th2) or TNF-α and IFN-γ (Th1). The mycelium of PhomaherbarumYS4108 contains polysaccharides that stimulate T cells via TLR2/4, improving immunological function. Polysaccharides from Ganoderma lucidum activate B cells, resulting in an increase in immunoglobulin synthesis. Neuroinflammation is reduced by fucoidan treatment, blocking the proinflammatory response of microglia (11). Additionally, the interaction between polysaccharides and the gut microbiota has garnered attention for its implications in immune health. Gut microbiota produces huge number of metabolites, consequently by metabolizing and anaerobic fermentation of complex polysaccharides. Short-chain fatty acids (SCFAs) may help maintaining immunological homeostasis and immune cell functions both locally and systemically. Polysaccharides improve antioxidant capacity, reduce the production of pro-inflammatory mediators, strengthen the intestinal barrier, influence the composition of intestinal microbial populations, and promote the synthesis of SCFAs (12). The potential of polysaccharides not only as direct immunomodulators but also as agents that can influence the broader immune landscape through gut health.

This review highlights the structural features, immunomodulatory properties, underlying immunomodulatory mechanisms of naturally occurring polysaccharides, and activities related to immune effects by elucidating a complex relationship between polysaccharides and immunity.

## 2 Polysaccharides from natural sources and structural diversity

**Figure 1:**
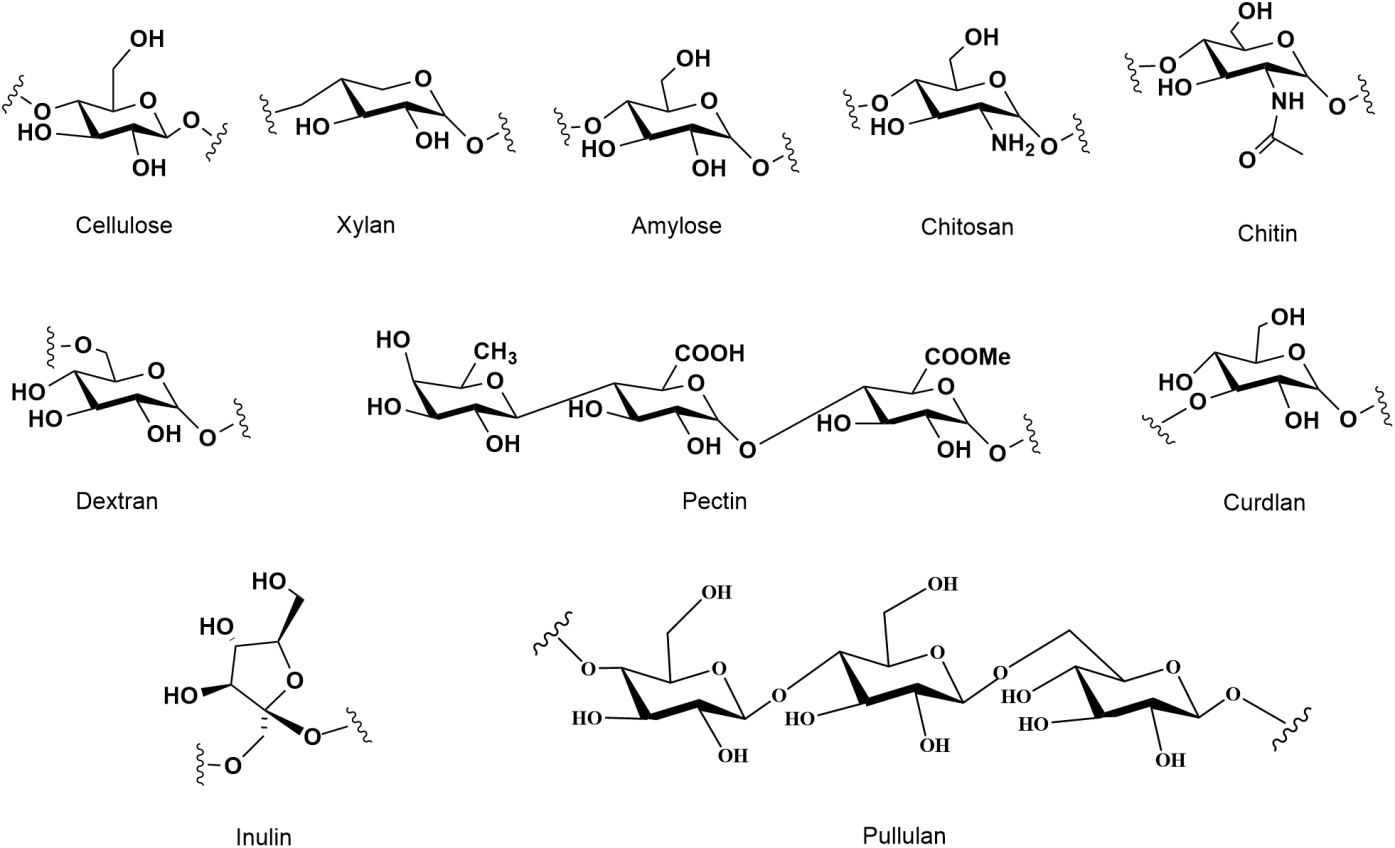
Types and structural arrangement of the common natural polysaccharides.

Polysaccharides are complex carbohydrates that play crucial roles in various biological processes across different organisms, including plants, animals, and microorganisms. Their structural differences significantly influence their biological activities, which can range from antioxidant properties to immunomodulatory effects. The molecular weight, number of branched chains, percentage of monosaccharides, and structural conformations of each polysaccharide source vary. As shown in **Figure 1**, types and structural arrangement of the common natural polysaccharides. Cellulose, starch, inulin and pectin are abundant in plant tissue. Animal polysaccharides include glycogen, chitosan and chitin. Glucan polysaccharides are widespread in mushrooms. Microbial polysaccharides include pullulan, xanthan gum, dextran, lentinan and curdlan. Exopolysaccharides are polysaccharides released by microorganisms. Polysaccharides from natural sources with structural diversity and different biological activities (**Table 1**). The structural differences among plant, animal, and microbial polysaccharides lead to distinct biological activities. Understanding these relationships is crucial for the development of polysaccharide-based therapeutics and functional foods, as their diverse structures can be tailored to enhance specific biological functions.

**Table 1:** Polysaccharides from natural sources with structural diversity and different biological activities.

Classifications	Natural source	Structural types	Structural characteristics	Biological activity	Reference
Plant	*Curcuma longa* L.	Cellulose	*β*-1,4-linked glucose	Antimicrobial	(13)
Plant	*Xanthosoma undipes*	Inulin	*β*-1,2-linked fructosyl–fructose	Prebiotics	(14)
Plant	*Enhalus acoroides* (L.f.) Royle	Pectin	*α*-1,4-linked D-galacturonan	Immunostimulation	(15)
Plant	*Codonopsis pilosula*	Pectin	arabinan and AG regions	Immunomodulatory	(16)
Plant	*Malus pumila* Mill.	Pectin	*α*-1,4-linked homogalacturonan	Immunomodulation	(17)
Plant	*Portulaca oleracea* L.	Pectin	*α*-1,4-linked homogalacturonan	Immunomodulatory	(18)
Plant	*Crataegus* spp.	Pectin	*α*-1,4-linked homogalacturonan	Immunomodulatory	(19)
Plant	Red seaweeds	Xylan	1,4-linked xylopyranose	Immunomodulation	(20)
Plant	*Glycyrrhiza*	Dextran	1,6-linked-glucan	Immunomodulatory	(21)
Plant	Lotus root	Dextran	1,6-linked-glucan	Immunomodulatory	(22)
Animal	Shrimp shells	Chitosan	Glucosamine and N-acetylglucosamine	Antimicrobial	(23)
Animal	Norway lobster	Chitin	N-acetylglucosamine	Antimicrobial	(24)
Animal	Sea cucumber viscera	Glycosaminoglycan	[→4)-β-D-GlcA-(1 → 3)-β-D-GalNAc- (1→]_n_	Immune-enhancing	(25)
Animal	Haliotis discus hannai Ino	Dextran	1,4-linked and 1,6-linked glucan	Immunostimulation	(26)
Animal	Sea urchin	Dextran	1,4-linked and 1,6-linked glucan	Immunomodulatory	(27)
Animal	Pleurotus	Dextran	*β-*1,3-linked and 1,6-linked glucan	Immunomodulatory	(28)
Microbes	*Ganoderma lucidum*	Dextran	*α/β*-(1, 3)/(1, 6)-glucan	Immune-enhancing	(29)
Microbes	*Poria cocos*	Dextran	*β*-(1-3)-D-glucan	Immunomodulatory	(30)
Microbes	*Poria cocos*	Dextran	1,3-linked-glucan	Immunomodulatory	(31)
Microbes	*Lactarius hatsudake* Tanaka	Dextran	*β-*1,3-linked and 1,6-linked glucan	Antioxidant	(32)
Microbes	*Paenibacillus* sp.	Curdlan	*β*-(1-3)-D-glucan	Immunomodulation	(33)
Microbes	*Aureobasidium pullulans*	Pullulan	*β*-1,3-1,6-glucan	Tumor immunology	(34)
Microbes	Lentinus edodes	Lentinan	*β*-(1-3)-D-glucan	Immunomodulation	(35)
Microbes	*Xanthomonas campestris*	Xanthan gum	1,4-linked glucan	Immunomodulation	(36)

### 2.1 Plant polysaccharides

Plant polysaccharides are primarily composed of cellulose, hemicellulose, and pectin, which are characterized by their linear and branched structures formed through various glycosidic linkages. Most plant polysaccharides are composed of glycosidic bonds such as *β*-(1→4)-D, *α*-(1→6)-D and *α*-(1→4)-D, which serve as links between various monosaccharides such as glucose, fructose and arabinose. A significant portion of cellulose, a biopolymer composed of chains of *β*-1,4-linked glucose units with a maximum length of millions of residues, is found in plant cell walls (37). Cellulose is the most important structural component of plants and is often used in the production of food, medicine and building materials (38). Furthermore, the combination of *β*-(1→2)-D-fructosyl-fructose bonds results in inulin, a diverse, water-soluble linear polymer (39). Inulin is an indigestible carbohydrate that occurs naturally in various forms, mostly as storage carbohydrates in plants (40). Pectin, the most complex polysaccharide found in plant cell walls, is mainly composed of branched rhamnogalacturonan segments (RG-I and RG-II) and linear 1,4-D-galacturonan segments (41). The diverse functional capabilities of pectin are influenced by its diverse structural features (42). According to research, plant polysaccharides are essential for immune modulation and other related diseases. *Platycodon grandiflorum* polysaccharide (PG) induced the phenotypic maturation of DCs, as evidenced by the increase in the expression of CD40, CD80, CD86 and major histocompatibility complex (MHC)-I/II on the cell surface (43). Furthermore, PG induces DC maturation by activating MAPK and NF-κB signaling downstream of Toll-like receptor 4 (TLR4). The structural diversity in plant polysaccharides is also enhanced by modifications such as sulfation and phosphorylation, which can significantly alter their biological activities (44).

### 2.2 Animal polysaccharides

Animal polysaccharides are very widespread and are found in almost all animal tissues and organs, especially in marine animals. Compared to the plant polysaccharides, animal polysaccharides, particularly glycosaminoglycans (GAGs), exhibit linear polyanionic structures that are crucial for various physiological functions. Chitin is the only positively charged natural polymer in nature that belongs to the straight-chain aminoglycans. Chitin consists of N-acetylglucosamine units (GlcNAc) with *β*- *(* 1, 4)-linked glycan, coupled with the presence of amino groups (at C-2) and hydroxyl groups (at C-3 and C-6) (45). Chitosan is a cationic polysaccharide formed by the deacetylation of chitin. Chitosan consists of N-acetyl-2-amino-2-deoxy-D-glucose (glucosamine, GlcN) and 2-amino-2-deoxy-D-glucose (N-acetyl-glucosamine, GlcNAc) monomer residues starting with *β* are linked (1→ 4) links (46). Chitin and chitosan activate peritoneal macrophages and NK cells to express a range of pro-inflammatory cytokines such as interleukin-1β (IL-1β), colony stimulating factor (CSF), and gamma interferon (IFN-γ) (47). Sulfated polysaccharides were isolated from sea cucumber viscera, which promoted the production of nitric oxide (NO) and cytokines (IL-1β, IL-6, and TNF-α) by RAW 264.7 cells and also their phagocytic activity through TLR4-mediated activation increased MAPKs and NF-κB signaling pathways (25). Animal-derived polysaccharides, have been shown to play essential roles in cell signaling and tissue repair. Their ability to modulate immune responses and promote wound healing is well-documented, with studies indicating that modifications in their structure can enhance these biological activities (48). For example, the introduction of sulfate groups in polysaccharides can confer anticoagulant and anti-inflammatory properties, making them valuable in therapeutic applications (49).

### 2.3 Microbial polysaccharides

Microbial polysaccharides, such as those derived from fungi and bacteria, display unique structural features that differ from both plant and animal polysaccharides. Some edible mushrooms like Ganodermalucidum, Poriacocos, and Ophiocordycepssinensis, contain polysaccharides, including glucans and heteropolysaccharides (50). In addition, among these polysaccharides there are large differences in molecular weight, type of chemical bonds and composition of monosaccharides (51). Over the last decade, observational studies have shown that polysaccharides from edible mushrooms have many biological effects, including regulating immunity, reducing obesity, and delaying aging (50, 52, 53). Ganoderma lucidum polysaccharides (GLPs) may include *α*/*β*-(1, 3)/(1, 6)-glucans and other carbohydrates with various biological properties. There is evidence that GLPs can alleviate obesity, enhance immunity, and treat colorectal cancers by affecting a specific gut microbiota that includes *Oscillopsia*, *Clostridium Cluster IV*, and *Eubacterium* spp (29). Polysaccharides found in Poria cocos consist mainly of the primary (1→3)-*β*-glucan and side chains of (1→6)-*β*-glucose. Poria coco polysaccharides (PCPs) possess anti-cancer properties by enhancing host immunity against cancer, directly leading to tumor cell death, and helping to defend against harmful biological stresses (54). The structural complexity of these polysaccharides allows for interactions with various biological targets, leading to enhanced immune responses and potential therapeutic benefits (55).

## 3 Structural properties of polysaccharides influence the ability of immune regulation

The influence of natural polysaccharides on the immune system has received more attention. The ability of polysaccharides to alter immunity depends primarily on the presence of unique structures such as branching regions, molecular weight, acetyl or sulfate groups, conformation, monosaccharide composition, and glycosidic linkages (56). The molecular weight of polysaccharides plays a crucial role, while lower molecular weight polysaccharides often show stronger immunomodulatory effects. Each polysaccharide has a significant molecular weight, and different compounds with different molecular weights affect immune regulation in different ways. Research found that the ability of Schisandrachinensis polysaccharides to alter the immune system is inversely correlated with their molecular weight (57). The reduced molecular weight and simpler structures of polysaccharides provide an advantage over other types of molecules and can easily pass through cell barriers (58). The effect of different polysaccharide molecular weights on immunity is complex and important. Immune stimulation: Low molecular weight polysaccharides typically have stronger immune stimulating effects. They have a greater ability to activate immune cells, including natural killer cells, dendritic cells and macrophages, which increases the production of cytokines and other signaling molecules involved in immunological responses (59). Pattern recognition: Low molecular weight polysaccharides often have simple structures that may be found more easily by the pattern recognition receptors (PRRs) of immune cells. This identification triggers innate immune responses and allows the adaptive immune system to mount a stronger defense against infection (60). Phagocytosis Activation: Low polysaccharides can improve the ability of immune cells to absorb and clear infections through a process called phagocytosis (61). This increases the overall effectiveness of the immune response and helps remove pathogenic organisms. Antiviral and antitumor activity: Some low molecular weight polysaccharides have been demonstrated to have potent antiviral and anticancer properties (62, 63). They can both directly stop the growth of tumor cells and viruses and strengthen the immune system for an intensive attack on diseases or cancer cells. Immunomodulation: Polysaccharides of different molecular weights can regulate immunological reactions in different ways. Smaller polysaccharides can have immunosuppressive effects, but larger polysaccharides typically promote immunological activation (63, 64). In diseases such as autoimmune diseases, which are characterized by dysregulated immune responses, this regulation can be used therapeutically.

In addition, branched polysaccharides tend to have higher immunomodulatory activity compared to linear ones. The highly branched side chains of pectin may influence the immunological regulatory effects of pectin on LPS-induced IL-6 production, as shown in *in vitro* studies (65). Research has shown that pectin can have an even stronger inhibitory effect on LPS-induced cytokine production when its rhamnogalacturonan I side chains are removed (66). At the same time, it was found that pectin polysaccharides with sulfate groups stimulated a number of neutrophil and macrophage effector functions (67). Standard polysaccharide moieties are structurally linked to cores that have linked chains or linked monosaccharide residues (56). Different functional groups and patterns trigger different immune responses, but sometimes high levels of acetylation result in impaired immune activity (68). Depending on their solution conformation, polysaccharides can interact with immune system cells or other components in different ways. These interactions can be single, triple or random coils. For example, the researchers found that the triple helix conformation promoted the production of TNF-α by immune cells, which enhanced the immunomodulatory effect on *β*-glucans (69).

The composition of monosaccharides in polysaccharides plays a crucial role in determining their biological activities, particularly their immunomodulatory effects. The immunoenhancing activity of a novel heteropolysaccharide isolated from custard apple pulp was closely related to its monosaccharide composition, particularly noting that specific monosaccharides like glucose and rhamnose can enhance immune responses in mouse macrophages and dendritic cells (70). Moreover, the specific types of monosaccharides present in polysaccharides can dictate their interactions with immune cells. The polysaccharides from *Ganoderma atrum* could induce anti-tumor activity through the activation of host immune responses, with the composition of monosaccharides such as mannose and galactose being pivotal in mediating these effects (71). Another study result further supports this notion, indicating that polysaccharides containing domains of specific monosaccharides like galactose and mannose exhibit significant immunostimulatory activity (72). Moreover, wang reviewed the effects of monosaccharide composition and proportion on the bioactivity of polysaccharides, emphasizing that variations in these parameters can lead to significant differences in their immunomodulatory effects (73). the monosaccharide composition of polysaccharides is a fundamental determinant of their immune activity. Variations in the types and proportions of monosaccharides not only influence the structural characteristics of polysaccharides but also their interactions with immune cells, leading to diverse biological effects.

Glycosidic linkages are fundamental to the structure and function of polysaccharides, which play crucial roles in various biological processes, including immune responses. The type and configuration of glycosidic linkages significantly influence the immunogenicity and biological activity of polysaccharides. The polysaccharides with *α*-1,6-linkages have been shown to enhance immune responses, as seen in studies involving α-glucans derived from microbial sources, which are essential for macrophage phagocytosis and the induction of innate immune responses through Toll-like receptor 2 (TLR2) pathways (74). The structural diversity of glycosidic linkages also contributes to the functional variability of polysaccharides. For example, the presence of different glycosidic bonds, such as *β*-1,3 and *β*-1,4 linkages in galacto-oligosaccharides, has been associated with various biological activities, including prebiotic effects and modulation of immune responses (75). Furthermore, polysaccharides derived from plants, such as those from *Radix Aconiti*, have demonstrated immunostimulatory effects, enhancing the host’s immune response and showing potential as biological response modifiers (74). Glycosidic linkages are pivotal in determining the immune activity of polysaccharides. The type and configuration of these linkages influence the immunogenicity of polysaccharides, their ability to activate immune responses, and their potential applications in vaccine development and therapeutic interventions. Scientists have found a variety of potential sites for the production of immune-regulating polysaccharides that could be used in functional foods. Hopefully, further studies on the structure-function relationships of immunomodulation including polysaccharides will be carried out soon. Overall, the structural diversity of polysaccharides plays a significant role in modulating immunoregulatory functions (76).

## 4 Immunomodulatory properties of polysaccharides

**Figure 2:**
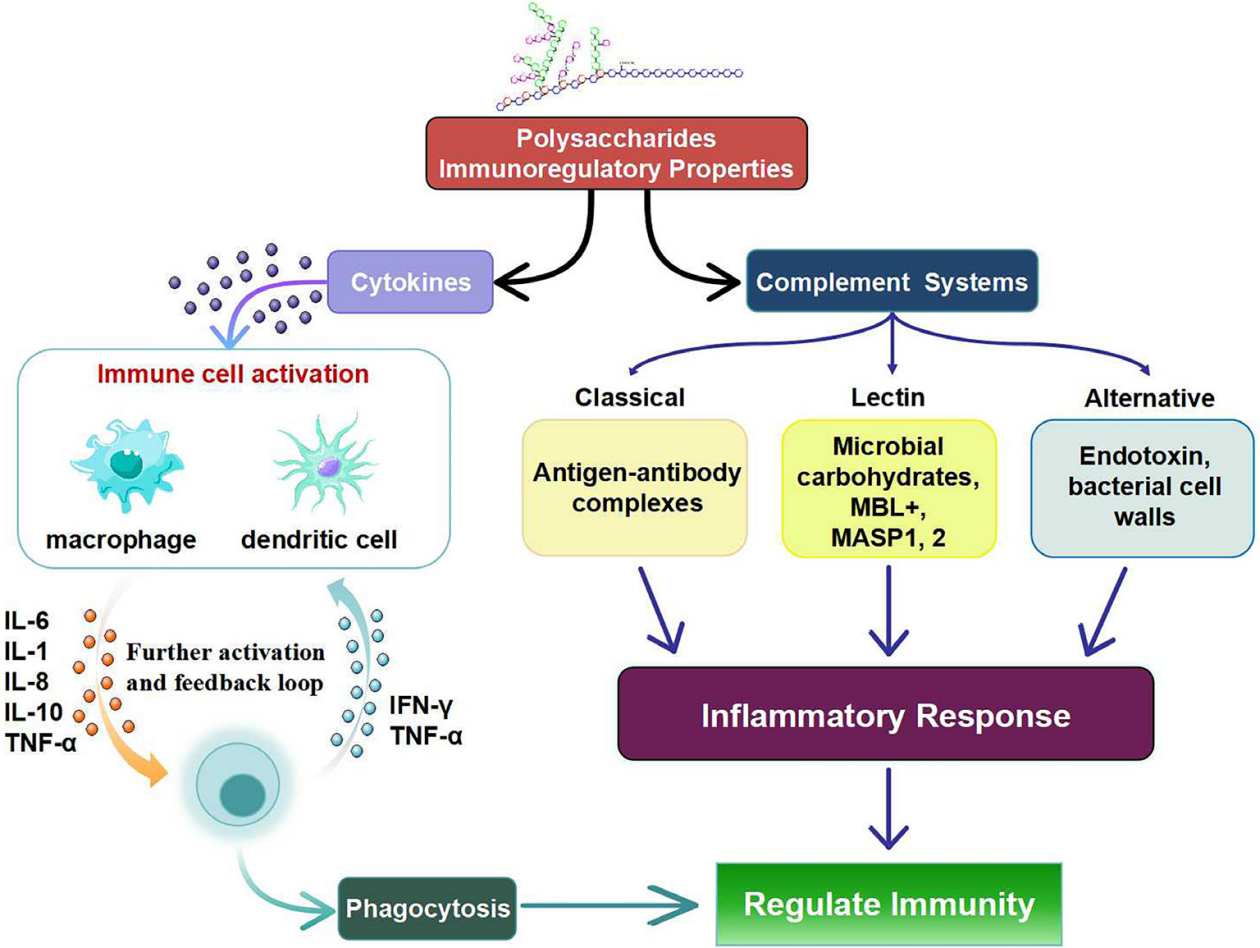
Immunomodulatory Properties of Polysaccharides via different cellular pathways.

Stimulating the complement system of dendritic cells and macrophages is the primary mechanism by which polysaccharides influence immunity (**Figure 2**). The increase in the number of lymphocytes and the immunological organ indices is within the scope of their possibilities. There are a number of signaling pathways and receptors on immune cells that also activate them (77). In addition to stimulating cytokines and complements, polysaccharides also stimulate the production of immune cells including macrophages, T cells, B lymphocytes, and (NK) cells, among others (78). Macrophages play a crucial role in the defense mechanisms of the host immune system (79, 80). In particular, polysaccharides influence the immunological responses of macrophages by increasing phagocytic activity, cell proliferation, ROS production and cytokine secretion (81).

Intercellular adhesions, cell division and cell proliferation are all regulated by cytokines. Interleukin (IL), colony stimulating factor (CSF), tumor necrosis factor (TNF), and interferons (IFN) are the four classes of cytokines. In addition to their essential functions in regulating inflammatory and immunological responses, polysaccharides also regulate adaptive and innate immunity (80). The polysaccharide component CPE-II can significantly increase the production of the pro-inflammatory cytokines TNF-α and IL-6 as well as the anti-inflammatory cytokine IL-12 in the macrophage cell line RAW 264.7 (82). Polysaccharides can simultaneously control the production of pro-inflammatory and anti-inflammatory cytokines. The fact that IL-12 acts as a negative feedback mechanism during the hyperinflammatory response to prevent overactivation of macrophages lends credence to the idea that the body has a self-regulatory system that keeps everything in balance. Likewise, cytokine production by macrophages can be induced by polysaccharides (83, 84).

Natural polysaccharides and cytokines can combine to stimulate the immune response of macrophages. Macrophages can release many cytokines such as NO, IL-1β and TNF-α when the polysaccharides SHP and IFN-γ work together. The transcript levels of the cytokines IL-1 and TNF-α also increased significantly. The synergistic effect also altered the differentiation markers CD11b, CD18 and CD24 produced by macrophages (85). Complement, through its numerous common activation pathways, plays an essential role in immunological control and the body’s response to microbial defense. Immunological effects, control of the complement system, and complement modulators for complement-related diseases are all aspects of sulfated glycosaminoglycans (86, 87). Fruits of the species *Capparis spinosa* L. contain sulfated polysaccharides that can inhibit both traditional and nontraditional pathways of complement activation, making them a promising candidate for therapeutic complement suppression (88). These processes include the many complement pathways, the mannose-binding lectin (MBL) pathway, and classical complement (89). Ca^+2^ is essential for the MBL protein. By binding to the mannose receptors of certain pathogen cells, lectins activate the MBL complement system, which in turn generates immunity (90).

## 5 Mechanisms of polysaccharide immune interactions

**Figure 3:**
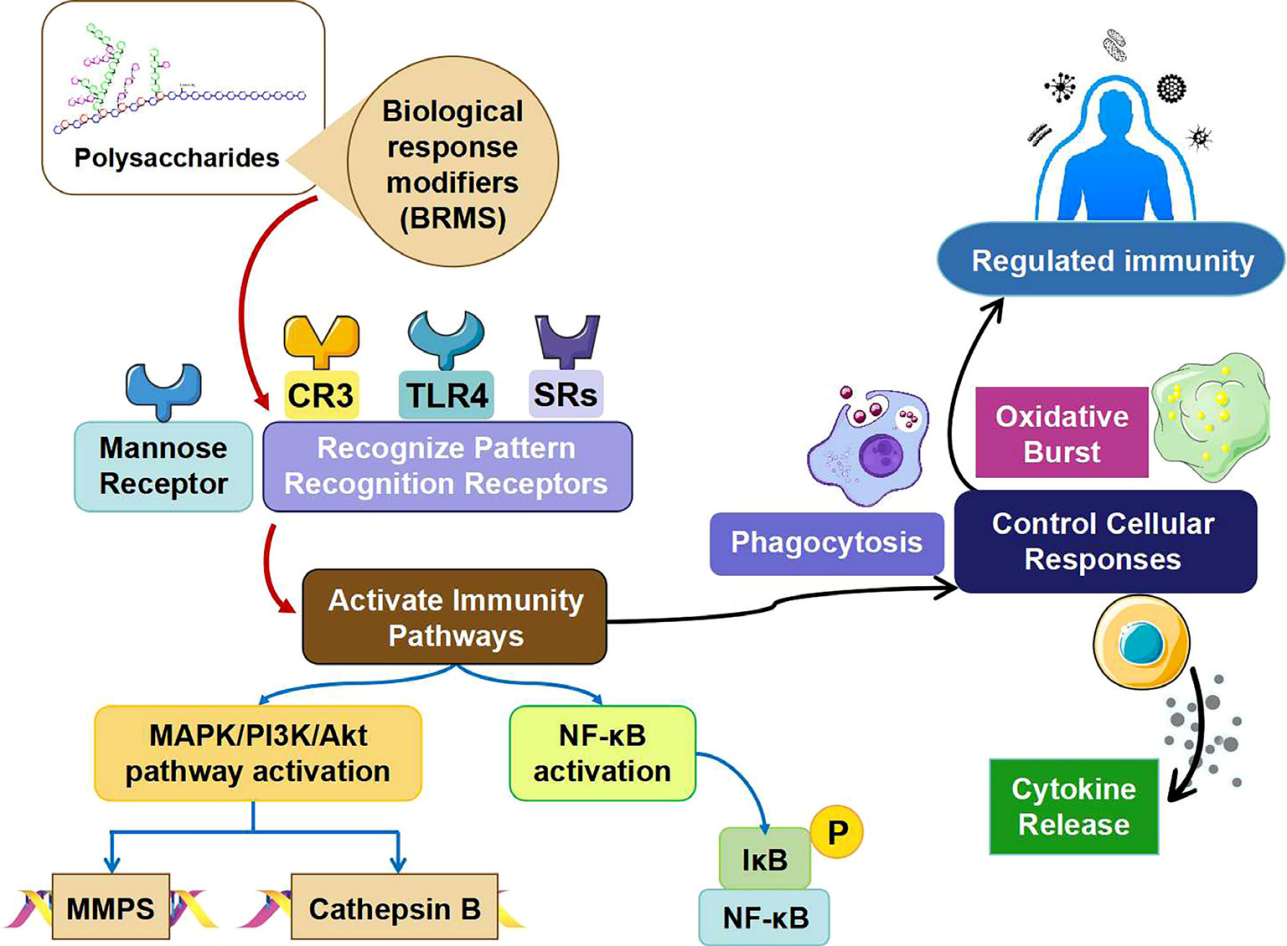
Different mechanism of action of polysaccharides in immunity responses and regulation.

Biological response modifiers (BRMs) are polysaccharides that possess immunomodulatory properties (**Figure 3**), allowing them to simultaneously influence innate and adaptive immune responses (91). Molecular recognition of polysaccharide BRM is achieved by proteins such as plasma proteins and pattern recognition receptors (PRRs). Pathogen-associated molecular patterns (PAMPs) are PRRs – nonclonal immune proteins – that identify common molecular features among different microbes. Beginning with their binding to PRRs, ligands activate genes of innate immunity through signaling cascades regulated by Rel and NF-κB (92).

Two different types of PRRs can be used for polysaccharide BRMs: scavenger receptors (SRs) and toll-like receptors (TLRs) (93). In order for TLRs to interact with intracellular signaling proteins and accept ligands, they have two unique features: the Toll/IL-1 receptor-like domain and leucine-rich repeats (94). Recognizing both endogenous ligands that support tissue homeostasis and immune defense signaling is the role of multidomain and multiligand receptors (SRs). Both macrophages and dendritic cells (DCs) produce SRs (94). The BRMs are identified by class A SR, β-glucan receptors, mannose receptors and complement receptor type 3 (CR3). Fucoidan is a polysaccharide ligand related to class A SR (95). A recognized binding site for Dectin-1,3-Glucan is the glucan receptor/Dectin-1 (96). The mannose receptor requires calcium for binding of mannosyl/fucosyl or GlcNAc glycoconjugate ligands (97). Both the CR3 membrane receptor and the adhesion molecule are essential. As previously mentioned, β-glucan, fixed iC3b and intercellular adhesion molecule-1 (ICAM-1) are the ligands that CR3 recognizes as a receptor (98).

The MBL of the lectin-complement pathway and proteins from the alternative and conventional complement pathway are among the proteins in blood plasma that can recognize polysaccharide BRMs. As a multimericlectin, mannose-binding lectin can detect multiple diseases without the need for a specific antibody (99). Activation of the lectin complement cascade occurs upon MBL attachment to macromolecules containing fucose, mannose, N-acetylglucosamine, and glucose residues that are aligned and densely packed like microorganisms. As a result, the body begins to respond by releasing inflammatory chemicals (100).

Polysaccharide BRMs can trigger specific cellular and molecular events and produce specific expression patterns depending on the receptors and binding proteins to which they bind. The receptors and binding proteins present in polysaccharide BRMs, including mannose receptors, CR3, β-glucan receptors, TLRs, SRs, lectin-binding pathway binding proteins, and the alternative and classical complement pathways, originate from innate immunity, as previously mentioned. Not only mast cells, neutrophils and epithelial cells express TLRs, but also myeloid cells (monocytes, macrophages and dendritic cells) (101). Two Toll-like receptors, namely TLR-2 and TLR-4, are known to recognize polysaccharide BRMs, out of ten Toll-like receptors found in humans (102).

Endothelial cells, DCs, smooth muscle cells, and macrophages all express SRs (103). To SR’s knowledge, fucoidan is one of the polysaccharide BRMs. Various cell types, such as B and T lymphocytes, neutrophils, DC, eosinophils and monocytes, express the *β*-glucan receptor (95, 104). Certain cell types, including DCs, hepatic endothelial cells, renal mesangial cells, macrophages, and tracheal smooth muscle, express mannose receptors (105). The mannose receptor is bound by the mannan-type polysaccharide BRM. Cells that primarily express CR3 are macrophages and neutrophils. Furthermore, CR3 is expressed by a tiny percentage of B and T cells as well as non-NK cells (106). Myeloid cells are the main targets of polysaccharide BRMs as they are essential for innate and adaptive immunity (107). One of the most important types of myeloid cells, macrophages, are stimulated by BRMs to increase their phagocytosis, ROS production, cytokine release and activation marker expression (such as FcR and B-7) (108, 109). As a result, polysaccharide BRMs improve effector function, antigen processing ability and regulation of acquired immunity by promoting cytokinesis, antigen presentation and expression of macrophage cell adhesion molecules. Therefore, polysaccharide BRMs can secondarily or consequently directly stimulate other immune cells such as lymphocytes and NK cells. In many cases, BRMs containing polysaccharides are very potent mitogens (80, 110).

Activation of the polysaccharide BRM leads to an increase in macrophages, NK cells, T lymphocytes and B lymphocytes. This happens, among other things, through the binding of polysaccharides to their receptors. Mitogen-activated protein kinases (MAPKs), involved in intracellular signaling events, are responsible for mitogen stimulation of macrophages (**Figure 3**), B lymphocytes, and NK cells by polysaccharide BRMs (111). Through their immunomodulatory function, BRMs promote the proliferation of immune cells and inhibit cell death (112). BRMs not only activate cells, they also stimulate the immune system’s complement pathways. One way the active complement system helps clear infections is by activating macrophages (113). Members of the *β*-glucan family comprise the polysaccharides with the greatest biological activity. Since they are PAMPs, glucans can be identified by various cell surface receptors on monocytes/macrophages, neutrophils, (NK) cells, DCs, T cells and B cells (114, 115).

In the presence of polysaccharides, interaction with CR3 leads to upregulation of NF-κB, activation of PI3K, and phosphorylation of Syk, the spleen tyrosine kinase. As a result of these events, cytokines such as interleukin IL-2, IL-10 and TNF-α are released (116). Various cells such as monocytes, macrophages, neutrophils, T cells and dendritic cells are known to express Dectin-1. *β*-Glucans promote innate immune responses (phagocytosis, reactive oxygen species production, IL-12, IL-6, IL-10 and TNF-α synthesis) by activating many signaling pathways such as Syk, Akt, MAPK and nuclear factor activated T cells (NFAT) (116, 117). Another type of receptor that binds polysaccharides and can identify β-1,3-glucans is lactosylceramide or (LacCer). LacCer expression is abundant in neutrophils, dendritic cells and macrophages. Together, polysaccharide and LacCer enhance the oxidative burst response of neutrophils, stimulate macrophage inflammatory protein-2, and activate NF-κB (118). Dendritic cells and macrophages have scavenger receptors that can identify fungal *β*-glucans. However, the exact mechanism by which these receptors are triggered is still unknown. It is generally believed that activation of the MAPK and PI3K/Akt kinases occurs through ligand interaction (119, 120).

## 6 Other roles of polysaccharides related to immunity

The immune activity of natural polysaccharides is closely related to intestinal immunity, anticancer, antigen recognition and presentation, antimicrobial and anti-inflammatory effects. As shown in **Table 2**, other effects of polysaccharides related to immunity. It not only helps maintain immune homeostasis and the body’s defense ability, but also provides a broad perspective for polysaccharides in the field of drug development and clinical application. The immune activity of natural polysaccharides allows the body to build a solid defense net.

**Table 2:** Other effects of polysaccharides related to immunity.

Resources	Related activity	Methods	Effects	Reference
Enteromorpha intestinalis	Intestinal immunity	*In vivo*	Promoting microbial growth, increasing in propionic acid content.	(121)
*Abrus cantoniensis* Hance	Intestinal immunity	*In vivo*	Increasing immune organ index and regulating secretion levels of immune cytokines. Improving intestinal morphology and increasing expression levels of tight junction proteins.	(122)
Glycyrrhiza	Intestinal immunity	*In vivo*	Increasing the production of sIgA and elevating Th1 and Th2 immune responses by facilitating the expression of IL-2, IL-4, IL-1β and IFN-γ. Increasing the proportion of CD4+ and CD8+ cells in the intestine.	(123)
*Callicarpa nudiflora* Hook	Intestinal immunity	*In vivo*	Affecting the expression of NF-κB/MAPK pathway-related proteins. Regulating intestinal flora and metabolism.	(124)
Huangshui	Intestinal immunity	*In vitro*	Inhibition of TRL4/MyD88/NF-κB and MAPK signaling pathways.	(125)
*Alhagi camelorum* Fisch	Intestinal immunity	*In vivo*	Promoted the secretion of serum lgG antibody, intestinal lgA antibody and intestinal cytokines, improved the morphology of intestinal villi and crypts, enhanced quantity of intestinal IELs and IgA+ cells, and activated T lymphocytes and DC cells in MLNs.	(126)
*Broussonetia papyrifera* leaf	Intestinal immunity	*In vivo*	Scavenging free radicals and activating the Nrf2 pathway, and enhancing antioxidant capacity. Reducing inflammation, and mitigating intestinal cell death.	(127)
Raspberry	Intestinal immunity	*In vivo*	Increasing the levels of Dubosiella and metabolite butyrate.	(128)
Penthorum chinense Pursh	Anticancer	*In vitro* and vivo	Inducing H22 cell apoptosis *in vitro* via the mitochondrial signaling pathway. Suppressing xenograft tumor growth by protecting immune organs, enhancing immune cells functionality, and inducing apoptosis.	(129)
*Lagotis brevituba* Maxim	Anticancer	*In vitro* and vivo	The inhibition of tumor cell proliferation *in vitro* may result in the inhibition of aerobic respiration and glycolysis. Inhibiting the expression of AMPK in tumors and enhance spleen function.	(126)
*Sanghuangporus* vaninii	Anticancer	*In vitro*	Facilitating the initiation of the immune reaction, and promoting the secretion of cytokines. Mediating the apoptosis of HT-29 cells by blocking them in S phase.	(130)
Paeonia lactiflora	Anticancer	*In vitro* and vivo	Suppressing the proliferation and migration of HepG2 cells. Enhancing the release of NO and cytokines (IL-6, IL-1β, and TNF-a).	(131)
Brassica rapa L.	Anticancer	*In vitro* and vivo	Increasing cytokine and NO production by macrophages in a Toll-like receptor TLR2 and TLR4-dependent manner.	(132)
Dendrobium officinale	Anticancer	*in vitro* and *in vivo*	Inducing apoptosis of HT-29 cells using the mitochondria-dependent intrinsic apoptotic pathway. Inhibiting tumor metastasis in the zebrafish model.	(133)
Lycium barbarum	Anticancer	*In vivo*	Exhibiting the prime effect on the macrophage repolarization, augmenting phagocytosis effect of the repolarized macrophages on breast cancer cells, and regression of breast tumor	(134)
Gum Arabic	Antimicrobial	*In vitro*	Three different S. aureus strains and two E. coli strains. Boost the antimicrobial activities of granulocytes and increasing intracellular ROS production, leading to more phagocytosis and intracellular killing.	(135)
*Akebia trifoliata* (Thunb.) Koidz stem	Antimicrobial	*In vitro*	Against Staphylococcus aureus, Bacillus subtilis, Salmonella, Penicillium italicum, Rhizopus and Aspergillus niger.	(136)
Ilex paraguariensis	Antimicrobial	*In vitro*	Against Gram-negative bacteria (Enterobacter cloacae, Salmonella enteritidis, and Salmonella typhimurium), Grampositive bacteria (Bacillus cereus, Micrococcus flavus, Staphylococcus aureus, and Listeria monocytogenes).	(137)
Enteromorpha prolifera	Antimicrobial	*In vitro*	Against bacillus subtilis, Staphylococcus aureus, Escherichia coli, Pseudomonas aeruginosa, Salmonella spp.	(138)
Sargassum fusiforme	Antimicrobial	*In vitro*	Against grampositive bacteria (S. aureus and B. subtilis) were stronger than those on gram negative bacteria (E. coli, P. aeruginosa and Salmonella spp.).	(139)
Antrodia cinnamomea	Anti-inflammatory	*in vitro*	Inhibiting TNF-α and IL-6 release. Reversing Iκ-B degradation induced by LPS in macrophages. Suppressing the AKT, and ERK signaling pathway.	(140)
*Alpinia zerumbet* (Pers.) Burtt. et Smith	Anti-inflammatory	*In vivo*	Regulating the NF-κB signaling pathway.	(141)
Scorias spongiosa	Anti-inflammatory	*in vivo*	Reducing IL-1β, IL-6, TNF-α, and IFN-γ and elevating IL-10.	(142)
Astragalus	Anti-inflammatory	*In vivo*	Increased the abundance of SCFAs-producing genus eg. *Oscillospira*, *Akkermansia*, and *Coprococcus*. Increasing the serum concentrations of SCFAs including butyrate and propionate, and their anti-inflammation effects were demonstrated on mice primary alveolar macrophages.	(143)
*Russula vinosa* Lindblad	Anti-inflammatory	*In vivo*	Scavenging the endogenous reactive oxygen species through the activation of Nrf2 and suppression of the NFκB signaling pathways.	(144)
*Smilax glabra* Roxb.	Anti-inflammatory	*in vitro*	Inhibiting NO secretion, reduce the levels of pro-inflammatory factors (IL-6 and TNF-α), and reduce LPS-stimulated inflammatory damage in RAW 264.7 cells by inhibiting activation of the NF-κB (p65) pathway.	(145)
*Ginkgo biloba*	Anti-inflammatory	*In vivo*	Regulating the expression of p-p65 p-IκBα, TNF-α and IL-1β proteins in the inflammation signaling pathway.	(146)

### 6.1 Intestinal immunity

The collection of bacteria that live and communicate in the human digestive tract is called the intestinal or gut microbiota (147). It is true that the microorganisms in our stomach have a remarkable ability to alter physiology in both healthy and sick states. Most human physiological systems are directly or indirectly influenced by intestinal bacteria. This includes the maturation of the immune system as well as metabolic and pathogenic processes (148). Due to their numerous biological applications, natural polysaccharides have gained interest and popularity as dietary nutrients in recent years. Polysaccharides, particularly those derived from dietary sources, have been shown to exert significant immunomodulatory effects through their interactions with gut microbiota and the metabolites produced therein. The mechanisms by which these polysaccharides influence immune responses are multifaceted and primarily involve the fermentation of indigestible carbohydrates by gut microbiota, leading to the production of SCFAs such as butyrate, propionate, and acetate. Natural polysaccharides can also reduce excessive inflammatory responses by altering the composition of the gut microbiota, promoting the production of SCFAs, strengthening the intestinal barrier, increasing antioxidant activity, and reducing pro-inflammatory mediators (149). These SCFAs play critical roles as signaling molecules that modulate host immunity by activating various G protein-coupled receptors (GPCRs) and other immune pathways (150, 151). Colonocytes rapidly absorb SCFAs during their production, usually via sodium- or H^+^-dependent monocarboxylate transporters. Examples of GPCRs to which SCFAs bind and influence intestinal mucosal immunity, barrier integrity and function include free fatty acid receptors 2 and 3, GPR109a/HCAR2 and GPR164 (152, 153). Additionally, SCFAs have been reported to enhance the production of antibodies in B cells by promoting metabolic pathways that support energy production and cellular function (154). SCFAs play an important role in maintaining intestinal immunological homeostasis. The invasion of pathogens could be prevented by triggering immune responses and removing antigens from neutrophils and monocytes (115, 155, 156). These SCFAs control a variety of functions such as: glucose homeostasis, inflammatory reactions, energy absorption and gastrointestinal motility. They are quickly absorbed by the large intestine (157).

Prebiotics are made from polysaccharides and serve various biological purposes, including maintaining intestinal flora. In addition to its beneficial properties, the intestinal microbiota selectively degrades polysaccharides so that it can use them as energy to maintain physiological effects and control the composition of intestinal bacteria (158). Certain polysaccharides, such as fiber cannot be hydrolyzed by the human stomach or small intestine. A chronically low fiber intake can lead to dysbiosis in the intestine and permanently change the microbiota there (159). Unlike fermentable polysaccharides, which are broken down and fermented to form a variety of metabolites that provide energy to the host, non-fermentable polysaccharides are secreted in the large intestine (160, 161). As some diseases progress, certain polysaccharides act as immunomodulators and regulate the immune response. In addition, natural polysaccharides can improve immunity by strengthening the function of immune cells and helpful bacteria (149, 162).

**Figure 4:**
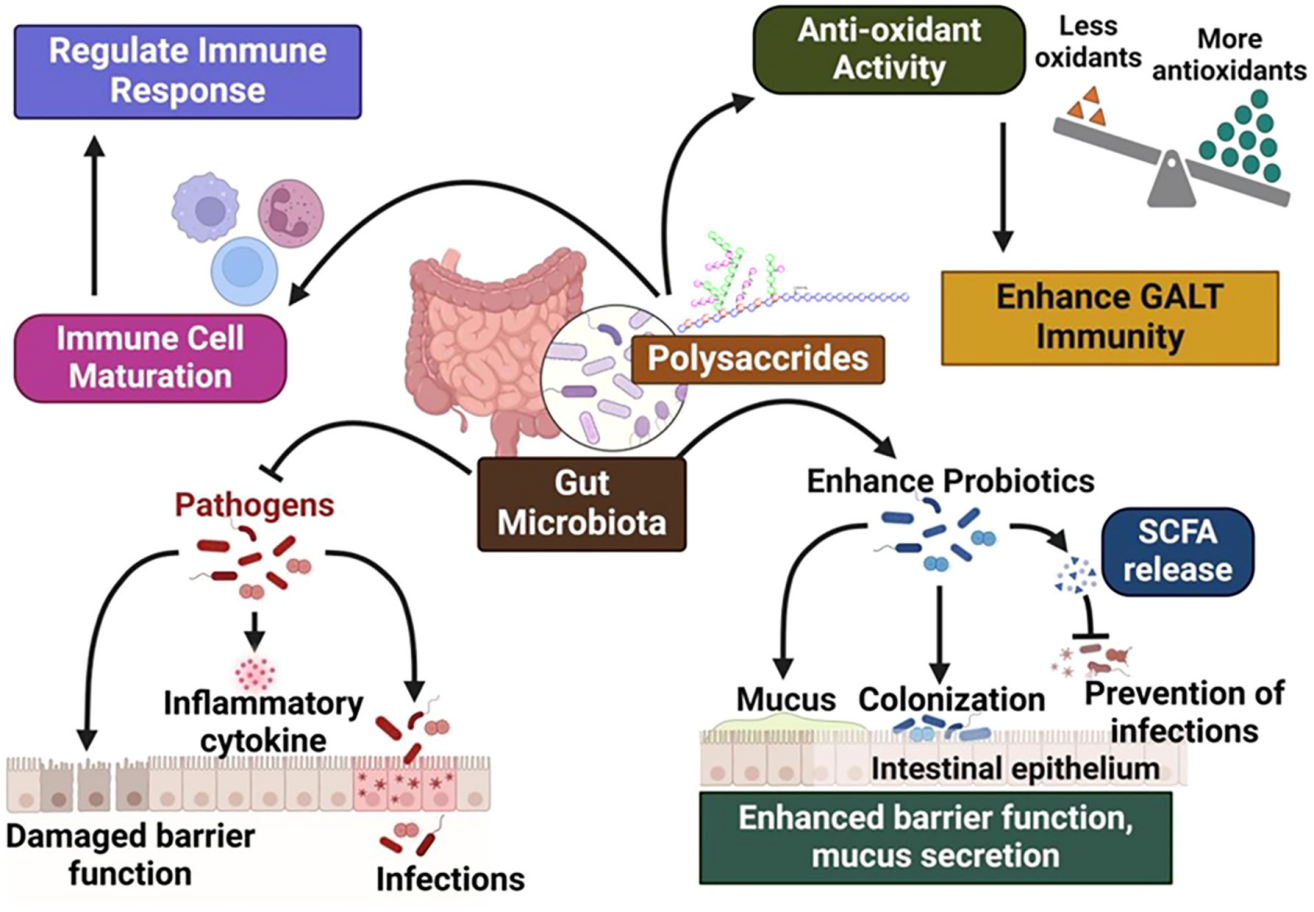
Dietary Polysaccharides role in Gut-Associated Immunity and GALT improvement.

Many polysaccharides, which come from natural herbs, mushrooms or yeasts, are used to treat intestinal disorders because they can repair damage to the intestinal lining (163). After an intestinal infection, free radicals accumulate in the gut-associated lymphoid tissue (GALT). This negatively affects mucosal and systemic immune function and rapidly reduces the antioxidant activity of intestinal tissue. Nevertheless, consumption of a large amount of bioactive carbohydrates from food can increase the antioxidant activity of intestinal tissue, thereby strengthening the host’s immunity (164). The main effector molecule in response to gastrointestinal mucosal immunity is secretory IgA. Numerous polysaccharides can enhance the response of T and B cells to antigens, which in turn increases IgA synthesis and ultimately strengthens the immunity of the digestive tract, thereby increasing the immunological activity of the adaptive system in GALT (1). Damage to the intestinal mucosa occurs more quickly when intestinal epithelial cells undergo abnormal apoptosis. Polysaccharides can prevent the death of endothelial cells (ECs) because they can block the Fas/FasL pathway and the synthesis of caspase-3 (165). Exopolysaccharides can enhance the phagocytosis of intestinal macrophages and slightly increase the production of nitric oxide (NO), thereby stabilizing the balance of the intestinal environment (41, 166). Part of the disruption of physiological intestinal function can be attributed to the infiltration of inflammatory cells into the intestinal tissue and their sustained activation. In order to repair the damaged mucosa, it is therefore imperative to induce apoptosis of the inflammatory cells. Polysaccharides have the ability to both up- and down-regulate the expression of Bcl-2, Bax and caspase-3 while dramatically reducing the formation of inflammatory bodies (41). As a result, the intestine experiences a variety of immunological responses, many of which are closely linked to the host’s systemic immunity. These considerations support the idea that homeostasis of the gut microbial community is necessary for GALT function. Therefore, the role of dietary polysaccharides in general gut-associated immunity and their help in GALT improvement is described (**Figure 4**).

Polysaccharides are essential for intestinal protection as they support intestinal integrity and well-being (127). They support the intestinal mucosal barrier, which acts as the body’s first line of protection from dangerous chemicals. Polysaccharides support the integrity of this barrier by increasing mucus production and promoting the development of tight junctions between epithelial cells, keeping infections, toxins and allergens out of the bloodstream. The polysaccharides from Callicarpa nudiflora Hook (CNLP) alleviated the clinical symptoms such as loss of body weight (BW), pathological damage and systemic inflammation by regulating the intestinal flora and its metabolism (124). In addition, polysaccharides have immunomodulatory properties that help regulate the immune response in the intestine. They have the ability to control the activity of immune cells, including that of macrophages, dendritic cells and lymphocytes, which helps maintain a healthy immune system and reduce gastrointestinal inflammation. The polysaccharide purified from *Alhagi camelorum* fish promoted the secretion of serum LgG antibodies, intestinal LgA antibodies and intestinal cytokines, improved the morphology of intestinal villi and crypts, increased the amount of intestinal IELs and IgA+ cells, and activated T lymphocytes and DC cells in MLNs (126). In diseases like inflammatory bowel disease (IBD) that are marked by intestinal inflammation, this anti-inflammatory action is very helpful.

Despite the promising findings regarding the immunomodulatory effects of polysaccharides, there are notable shortcomings in the current research landscape. One significant limitation is the variability in the structural characteristics of polysaccharides, which can lead to inconsistent biological effects (167). Furthermore, the precise mechanisms through which different polysaccharides exert their effects remain poorly understood, necessitating further investigation into their bioavailability and the specific metabolic pathways involved (168, 169). Future research should focus on elucidating the complex interactions between polysaccharides, gut microbiota, and host immune systems, potentially employing advanced metabolomic and metagenomic approaches to track the effects of specific polysaccharide structures on microbial metabolism and immune modulation.

### 6.2 Anticancer

Polysaccharides have demonstrated potent anticancer activity against a range of cancer cell types. Their minimal dangerous side effects and their selectivity against tumor cells make them a viable replacement for current chemotherapeutic cancer drugs. Most polysaccharides derived from plants, microbes, fungi and marine sources have been discovered to cause apoptosis in cancer cells. Their mechanisms of action include DNA damage, cell cycle arrest, rupture of the mitochondrial membrane, and the production of nitric oxide, which kills cancer cells and prevents them from spreading (170). The mode of action of some polysaccharides was evaluated through *in vitro* research on cell lines, and the effectiveness of other polysaccharides was assessed through *in vivo* research on appropriate animal models (129, 171, 172). The most commonly observed mechanisms were immunomodulation, cell cycle arrest, and depolarization of the mitochondrial membrane nitric oxide pathway. Recent studies have shown that polysaccharides can inhibit tumor growth by stimulating the immune system, thereby promoting immune cell proliferation, increasing cytokine secretion, and regulating immune functions throughout the body. Lachnum polysaccharide led to the accumulation of anti-tumor immune cells and reduced the infiltration of immunosuppressive cells such as myeloid-derived suppressor cells (MDSCs) and Treg cells, thereby enhancing anti-cancer immunity (173). *Sanghuangporus vaninii* polysaccharides (SVPS2) could facilitate the initiation of immune response, promote the secretion of cytokines, and mediate the apoptosis of HT-29 cells by blocking them in S phase *in vitro*. The antitumor mechanism of SVPS2 may be associated with an enhancement of the immune response (134). Therefore, polysaccharides can improve the body’s immune functions and play an immunomodulatory role by activating the upstream immune cells and promoting the production of cytokines, thereby inhibiting the growth of tumors (174).

### 6.3 Antigen and antimicrobial

The most important causes of disease worldwide include encapsulated bacteria such as Neisseria meningitidis, *Haemophilus influenzae serogroup* B (Hib) and *Streptococcus pneumoniae*. Because disease-preventing anticapsular antibodies often provide protection, efforts to develop vaccines against these pathogens have focused on their capsular polysaccharides (CPS) (175). When administered to newborns or some immunocompromised individuals, the capsular polysaccharide immunizations currently available against these diseases are neither immunogenic nor protective. Polysaccharide antigens generally trigger a T-independent immune response, which is insufficiently immunogenic and lacks memory in extreme situations of life (176). Conjugate vaccines were developed to address the poor immunogenicity of CPS vaccinations. These antigens can trigger a T-dependent immune response by conjugating CPS to carrier proteins (177). The development of technical experiments to produce nanoparticles that can transport antigens is currently the focus of great attention worldwide. Vaccinations have become significantly safer and more effective thanks to polysaccharide nanoparticles. The use of these biopolymers has been shown in previous research to enhance the immune response, reduce side effects, accelerate immunomodulatory activity, and maintain antigens in an expanded and regulated state by encapsulating them in polysaccharides (178). Extensive testing has been carried out with polysaccharides, particularly lactic glycolic acid. However, issues with biocompatibility and biodegradability led researchers to conclude that innovative biomaterials should be used in vaccine development. Consequently, antigen engineering has recently dominated studies using natural polysaccharides. In addition to their influence on biofilm formation, cell walls, cell membranes, nucleic acids, mycoproteins and intracellular metabolic pathways, polysaccharides should also be investigated for their possible antibacterial potential. Polysaccharides can inhibit the antibacterial effects of other bioactive chemicals (135, 136). The antimicrobial peptide increases intracellular ROS, leading to intracellular DNA degradation and cell death. Stimulating the cell’s production of antimicrobial peptides and improving the cell’s immunity could potentially be how polysaccharides work as antibacterial agents (179, 180).

### 6.4 Anti-inflammatory

At the end of the last century, there was great interest worldwide in researching the anti-inflammatory and immunomodulatory potential of polysaccharides (181). Wang et al. successfully isolated a sulfated polysaccharide from brown algae (*Sargassum cristaefolium*) and demonstrated anti-inflammatory effects in LPS-exposed RAW 264.7 cells (182). The polysaccharide in sea buckthorn (*Hippophae rhomboids* L.) berries can protect the liver of mice from CCL4-induced damage by reducing inflammation through antioxidant effects (183). Research has shown that cytokines, including TNF-α as well as IL-1β and IL-6, have a significant impact on pro-inflammatory responses. Endothelial cells lining blood vessels used the adhesion molecules produced by inflammatory cells to attract monocytes, lymphocytes and neutrophils. These cells traveled from the blood vessel to the damaged tissue, where they remained and caused hypotension, necrosis and death, symptoms similar to sepsis (184, 185). One approach to treating inflammation is to either prevent the generation of inflammatory mediators or ameliorate the dysregulation of proinflammatory (IL-1β, IL-6, and TNF-α) and anti-inflammatory (IL-10) cytokines. Certain anti-inflammatory medications work in the following ways. Triggering of inducible nitric oxide synthase (iNOS) enables massive NO production in response to pro-inflammatory cytokines, LPS, bacteria or viruses. Among the many indicators of inflammatory diseases and inflammation, the molecule NO stands out. Inflammatory processes are also influenced by cyclooxygenase (COX). Activation of COX-2 and iNOS leads to the production of several mediators that promote inflammation (186, 187). Suppressing or downregulating their expression is more effective than reducing the severity of the inflammatory response (188). Purple sweet potatoes were used to extract a dilute alkali-soluble polysaccharide (ASPP), which was then purified using a Sephadex G-200 column with DEAE-52 cellulose. NO production in RAW264.7 cells was induced by LPS. The anti-inflammatory properties of ASPP were examined using a mouse model and seven different macrophage cell lines (189). Scientific studies have shown that ASPP can improve IL-10 synthesis by RAW 264.7 cells. In a dose-dependent manner, 7 types of alternatively activated macrophage cells were activated by LPS and simultaneously decreased the levels of NO, IL-6, IL-1β and TNF-α. Furthermore, mice showed reduced levels of IL-1β, IL-6 and TNF-α expression after ingesting LPS. Additional sulfated homopolysaccharide was also formed by flesh of Cipango paludinachinensis (CCPSn). The ratios of pro-/anti-inflammatory cytokine secretion such as TNF-α/IL-10, IL-6/IL-10 and IL-1β/IL-10 were significantly reduced as this chemical inhibited the expression of COX2 and NOS. A reduction in the release of nitric oxide (NO) and prostaglandin E2 (PGE2) was also noted (190, 191). The use of natural polysaccharides from Pleurotus eryngi (PEPS) significantly and dose-dependently reduced the cytokine secretion ratios for IL-1β/IL-10, IL-6/IL-10 and TNF-α/IL-10 according to the research of Li et al. (192).

## 7 Conclusions

In conclusion, polysaccharides are vital biomacromolecules involved in many important biological activities. They have attracted great scientific interest due to their health benefits, including immune stimulation and immunomodulation, associated intestinal immunity, anticancer, antigenic, antibacterial and anti-inflammatory activities. The wide variety of polysaccharides found in nature is due to the many different species of plants, animals and microbes. Polysaccharides provide a rich source of bioactive compounds with significant potential for health and medicine. Their non-toxic nature and significant influence on biological functions, especially on immunological cells, make them valuable for the treatment and prevention of diseases. Polysaccharides hold promise as modulators of the host’s defense and immune systems, and their immunoregulatory functions are highly dependent on their structural features. Polysaccharide structure, molecular weight, acetyl or sulfate groups, and branching areas greatly influence how they interact with immune cells and other components of the immune system. In addition, polysaccharides are essential for gut-associated immunity as they modulate inflammatory responses, improve the integrity of the intestinal barrier, influence the composition of the gut microbiota, and promote the formation of SCFAs. Due to their ability to alter immune responses, induce apoptosis, and reduce tumor cell proliferation, polysaccharides have shown promise as potential anticancer drugs in cancer immunotherapy. Polysaccharides also have antimicrobial properties. They prevent bacteria from multiplying by preventing them from forming biofilms, forming cell walls and blocking their intracellular metabolic processes. By regulating the secretion of anti-inflammatory cytokines, limiting the expression of inflammatory mediators, and modifying the release of pro-inflammatory cytokines, they can also exert anti-inflammatory effects. Overall, polysaccharides represent a broad class of chemicals that have important implications for immunological function, host-microbiota interactions, cancer immunotherapy, antimicrobial defense, and inflammatory modulation. Further exploration of the structure-function relationships of polysaccharides and their medical applications promises to improve our understanding of immunology and develop new approaches to treating immune-related diseases.
